# Mentalization-Based Parenting Program for Child Maltreatment Prevention: A Pre–Post Study of 12-Week Lighthouse Group Program

**DOI:** 10.3390/children10061047

**Published:** 2023-06-12

**Authors:** Lina Gervinskaitė-Paulaitienė, Gerry Byrne, Rasa Barkauskienė

**Affiliations:** 1 Institute of Psychology, Vilnius University, LT-01513 Vilnius, Lithuania; rasa.barkauskiene@fsf.vu.lt; 2 Department of Social Policy and Intervention, University of Oxford, Oxford OX1 2ER, UK; gerry.byrne@spi.ox.ac.uk

**Keywords:** child maltreatment prevention, mentalization-based intervention, parent group, parenting program, prevention of adverse childhood experiences

## Abstract

The aim of this study was to assess the 12-week group version of the mentalization-based Lighthouse Parenting Program for child maltreatment prevention. Parents who might be facing mentalizing difficulties due to challenges in the parent–child relationship were invited to participate in the program. The aim of the program was to promote mentalizing—to encourage parents’ curiosity about their children’s minds and their willingness to reflect on their own feelings, thoughts, and behaviors. Study participants were 101 parents (82 mothers, 19 fathers). Parenting practices and parental and family adjustment were assessed using the Parent and Family Adjustment Scale and mentalization was measured using Mentalization Scale at pre- and post-intervention assessments. Parents’ feedback on the program was gathered after the program. Results revealed that mentalization, parental adjustment, and family functioning improved while coercive parenting practices decreased after the intervention. Study results provide preliminary indications of the benefits of the 12-week Lighthouse Parenting Program for parents referred or self-referred for mental health services due to their own or their child’s difficulties.

## 1. Introduction

Adverse childhood experiences (ACEs), in particular, childhood maltreatment, is one of the major risk factors for a variety of negative developmental consequences, such as emotional or behavioral problems in childhood [1] and adulthood [2]. Therefore, the prevention of ACEs is an important objective in the global and public health and family welfare fields [3,4]. Research shows that parenting is a significant factor in child maltreatment [5]. Difficulties in parents’ own adjustment and functioning can increase the risk of disturbed parenting and child abuse [6]. When parents become unable to handle the burdens of childcare or experience high levels of stress or depressive tendencies, the risk for children to experience ACEs increases substantially [7]. In addition, current psychological problems in children might be associated with increased parental stress, which is a risk factor for maltreatment [8] and might also indicate current or pre-existing challenges in parenting or impaired parent–child relationships. Thus, focus on parenting in the prevention of ACEs is a highly important strategy [9,10].

Parenting programs are widely used in the prevention of child maltreatment [11,12]. Meta-analytic findings demonstrate that parenting programs are effective intervention approaches for preventing child maltreatment from post-test to follow-up periods; they work by reducing problems such as parents’ inappropriate attitudes toward child rearing, abusive parenting behaviors, insufficient parenting skills, and insensitivity; at the same time, their strengths-based approaches enhance protective factors [13]. Despite this, the findings from the same meta-analysis reveal that parenting programs were not very effective in reducing parental depression and stress. In addition, an umbrella review by van Ijzendoorn and colleagues [5] shows that even though child maltreatment interventions are effective, they are of modest effectiveness. Thus, in this context, there is still a need for the development or expansion of parenting programs in efforts to prevent adverse childhood experiences through also addressing parental mental health and/or stress as the potential sources of risk for child maltreatment.

In recent years, one of the most promising approaches in parental support is the focus on the impact of mentalization in the parent–child relationship [14]. Mentalizing is the capacity to imagine and understand mental states in the self and other such as desires, needs, emotions, beliefs, goals, and thoughts and to perceive these as underlying behavior [15,16]. It is a core component of the child–parent relationship [17] and allows parents to remain emotionally regulated during times of difficulty [18]. Despite its protective potential, mentalizing itself can be compromised in times of high emotional arousal, which can often occur in parenting which is known to be stressful, and in such moments stress can overwhelm and lead to significant and frequent lapses in mentalizing for most people [19]. Loss of mentalizing or impaired mentalizing can also be related to biased interpretation or non-recognition of a child’s motives or psychological needs and thus, in this way, may contribute not only to disturbed parenting but to a pattern of emotional or physical neglect [20]. These also can be considered adverse environmental hazards, marking the lack of optimal circumstances to children’s adaptive and healthy development [21]. Therefore, interventions aiming to increase mentalization are theorized to have a multitude of beneficial effects for parent and child, and in particular for parent and child relationships under conditions of stress [20]. There has been a rise in the use of mentalization-based interventions for parents, as well as an increase in the amount of research being conducted on these interventions [19,22,23,24]. The latest systematic review [19] collected the available evidence for group-delivered mentalization-based parenting interventions and identified 10 relevant studies that examined six different interventions across a variety of populations. It was found that eight of the ten studies reported significant improvements in parental mentalization following the intervention. This is in line with the systematic review [22], which found tentative support for mentalization-based treatments with children and families, specifically in increasing mentalizing levels. Although the available data are promising, more studies are needed to better understand the effectiveness and potential benefits of these interventions. 

The Lighthouse Parenting Program (LPP) is an adaptation of mentalization-based treatment (MBT) with a specific focus on parental mentalization. It was initially developed for parents whose children are at risk for maltreatment [20]. The program aims to strengthen parent–child relationships, increase sensitive parenting, and enhance parents’ capacity to better understand their children. The LPP combines psychoeducational elements and group therapy processes [20]. To help parents understand mentalization and attachment processes more easily, metaphors and images are used in the program. The central metaphor is of a parent as a lighthouse, providing an illuminating beam (mentalizing) for thechild and guiding the child back to the safe harbor for support and comfort when the child needs it. In addition to the original version [20], LPP is being adapted for use with different populations—e.g., parents receiving inpatient psychiatric treatment [25] and families receiving mental health services [26]. Different versions are being evaluated regarding their effectiveness in randomized controlled trials (RCTs) in the United Kingdom [27], Germany [28] and Denmark. 

In the current study in Lithuania, we assessed the 12-week group version of the Lighthouse Parenting Program [26] for parents who might be facing mentalizing difficulties due to challenges in the relationship with their child(ren) because of their own mental health difficulties and/or the child’s psychological difficulties or problematic behavior. The clinical and scientific interest in LPP is growing and there is a strong need to expand the evidence base of the program’s benefits for different groups of parents. The current study is the first one which provides data for the 12-week group-based LPP version. Thus, we aimed to evaluate the preliminary effectiveness of the program in reducing problematic aspects of parenting, increasing positive parental relationships with children and parental mentalization as a protective factor. For parenting factors, we hypothesized that coercive parenting would decrease, and positive encouragement, parental consistency, and the parent–child relationship would improve after the intervention. In terms of parental and family functioning, we expected that parental emotional adjustment, family relationships, and parental teamwork would increase after the program. We also expected that mentalization would improve after the intervention. In addition, we aimed to analyze how parents evaluated the program, what experience they had in the program, and what was important to them in the program. 

## 2. Materials and Methods

### 2.1. Participants

This was a one-group pre–post intervention study which was pre-registered [29]. 

The inclusion criteria for participants were as follows: (a) parents with at least one child aged 12 or younger; (b) parents themselves or their child had been referred or self-referred for mental health, psychosocial, or psychoeducational services due to their own mental health problems, interpersonal difficulties, family issues, child rearing problems, and/or emotional, behavioral, or developmental problems of their child; (c) parents spoke Lithuanian; and (d) parents agreed to participate in the study (to attend the parenting group and fill in questionnaires before and after the group sessions). Exclusion criteria were current mental health needs that needed to be addressed in inpatient treatment (e.g., psychotic breakdown, severe depression, etc.), significant intellectual disability, other needs or problems incompatible with the parenting group intervention format, and/or participation in other continuing parenting groups.

Study participants were parents, with at least one child aged 12 or younger, who chose to participate in one of the Lighthouse parenting groups and agreed to fill in the study questionnaires. Informed consent was obtained from all the participants in the study. The program was recommended to parents by the specialists working with families, parents, or children, or parents self-referred.

All parents (*n* = 101) who started the program were included in this study (82 mothers and 19 fathers aged 24–60 years (M = 39.18, SD = 5.86)). Most of the parents lived in urban areas (94%) and the majority had higher education (89%), 8.9% had secondary education, and 1 participant had lower-than-secondary education. Regarding marital status, 78% of parents were married or cohabitating, 10% were separated/divorced, 8% were single, 2% were widowed, and 2% were in a long-term relationship, not cohabitating. As for occupational status, 81% of the parents were employed, 6% (all mothers) were on parental leave, 10% were unemployed, and 3% were college or university students. Just over half of the participants (54.5%) had 2 children, 23% had 1 child, 18% had 3 children, and 5% had 4 children. It is important to note that 73% of the participants reported having experience of seeking mental health services, and 44% had been receiving some kind of mental health services at the time they started the group. In addition, 73% had sought professional services for their child’s mental health or developmental problems, and 45% had children who were receiving services when the parents started attending the group.

Recruitment: Parenting groups were organized in collaboration with centers for psychological/psychoeducational services and one non-governmental institution providing a variety of services for children and families. Participants were recruited through these institutions; invitations to the study were also shared publicly through different websites related to parenting, online parenting groups, mailing lists, etc. Interested participants were invited to register for the study and attend an initial screening interview to check that they were eligible for the intervention (see inclusion criteria). 

### 2.2. Data Collection

All participants filled in the informed consent forms before participating in the study. The study was approved by the Vilnius University Psychological research ethics committee. Both assessments (baseline and post-intervention) were carried out online. At baseline, participants received the link to the online questionnaires and filled them in before the individual introductory session of the program. After the intervention, participants received the personalized link for the post-intervention questionnaires from the researchers. 

### 2.3. Intervention

The Lighthouse Parenting Program aims to promote parental mentalizing—to encourage parents’ active curiosity about their children’s minds and their willingness to reflect on their own feelings, thoughts, and behaviors [20]. In this study, a 12-week manualized mentalization-based parenting program [26] was delivered in group sessions. Before the start of the groups, all participants had an individual introductory session with one of their group facilitators. A total of 12 group sessions were carried out once a week; each session lasted around 2 h, including a break. Over these 12 sessions, facilitators introduced the topics of mentalizing and attachment using Lighthouse metaphors and psychoeducational materials (short presentations, videos) and combined them with experiential activities, group discussions, and MBT group therapy processes. Facilitators followed the program’s manual in introducing the topics over the course of the program and they could choose specific activities for each session from the ones suggested in the manual. Parents also received printed psychoeducational materials which summarized the information about metaphors (e.g., illuminating beam for mentalizing, safe harbor, parent as a lighthouse, etc.) used in the program. 

At least two facilitators were required to lead a group. All facilitators were qualified psychologists who had received Lighthouse program facilitator basic training from the program author. The training covered the main principles and main modules of the Lighthouse Parenting Program, the use of the program manual, the importance of mentalizing in parent–child relationships, core mentalization-based treatment (MBT) competences, and Lighthouse-specific competences. Part of the training was experiential; facilitators had a chance to take part in several exercises from the program to better understand what it might be for parents to participate in them and to have experience of the MBT stance and skills themselves. Training also consisted of interactive discussions, role plays, and group reflection sessions. Weekly group supervision was provided for facilitators by the program author and by another Lighthouse supervisor during the study. 

The program was delivered in organizations providing mental health and psychosocial services. In total, there were 9 groups with 7–14 parents in each group. 

### 2.4. Measures

#### 2.4.1. Parenting Assessment

Parenting practices and parental and family adjustment were assessed using the Parent and Family Adjustment Scale (PAFAS) [30]. The inventory consists of two scales with 30 items in total. The Parenting Scale (18 items) encompasses 4 subscales on parenting practices and the parent–child relationship: Coercive Parenting (5 items, e.g., “I shout or get angry with my child when they misbehave”), Positive Encouragement (3 items, e.g., “I praise my child when they behave well”), Parent–Child Relationship (5 items, e.g., “I have a good relationship with my child”), Parental Consistency (5 items, e.g., “I give my child what they want when they get angry or upset”). The 12-item Family Adjustment Scale encompasses 3 subscales and includes 3 domains of adjustment: Parental Adjustment (5 items, e.g., “I cope with the emotional demands of being a parent”), Family Relationships (4 items, e.g., “Our family members fight or argue”), and Parental Teamwork (3 items, e.g., “I work as a team with my partner in parenting”). The latter subscale applies only to respondents who are in a relationship. Respondents rate each item on a 4-point Likert scale from “Not true of me at all” (0) to “True of me very much” (3). Some items are reverse scored. For each subscale, the items are summed up to provide scores, with higher scores indicating higher levels of dysfunction, e.g., a higher score in Positive Encouragement indicates less-frequent use of positive encouragement, a higher score in the Parent–Child Relationship subscale indicates a poorer parent–child relationship, etc. The Lithuanian version of the PAFAS was prepared using back translation, which was reviewed by the authors of the scales. In this study, the Cronbach’s alphas for most of the subscales in the Parenting Scale showed good internal consistency: Coercive Parenting α = 0.71, Positive Encouragement α = 0.72, and Parent–Child Relationship α = 0.85. Only the Parental Consistency subscale showed low internal consistency, with a Cronbach’s alpha of 0.38. Consequently, we did not use this scale for further analysis. All the subscales in the Family Adjustment domain showed good internal consistency—Parental Adjustment α = 0.80, Family relationships α = 0.78, and Parental Teamwork α = 0.79.

#### 2.4.2. Mentalization Assessment

Parental ability to mentalize was assessed using the Mentalization Scale (MentS) [31]. It is a 28-item self-report measure that consists of three underlying dimensions: self-related mentalization, other-related mentalization, and motivation to mentalize. The items are rated by respondents on a 5-point Likert scale by evaluating how correct the item is for the respondent (from 1—Completely Incorrect to 5—Completely Correct). Examples of items are “I am often confused about my exact feelings”, “When someone annoys me I try to understand why I react in that way”, and “To understand someone’s behavior, we need to know her/his thoughts, wishes, and feelings”. The overall score of mentalization capacity is obtained by summing up all items, with higher scores indicating higher levels of ability to mentalize. The Lithuanian version of the scale was prepared with back translation and reviewed by authors of the scale. In this study, the scale showed good internal consistency with a Cronbach’s α = 0.86. 

#### 2.4.3. Parents’ Evaluation of and Feedback on the Program

In the post-intervention assessment, we asked two closed and two open questions about parents’ experience, opinion, and evaluation of the program. We included two Likert-type questions about the parents’ evaluations:

1. How do you rate the benefits of the program you have attended? Participants were asked to choose the most suitable rating on a Likert scale from 1, which meant “Not at all useful”, to 7, “Very useful”.
2. How likely are you to recommend it to other people? Participants were asked to choose the most suitable rating on Likert scale from 1, which meant “Would definitely not recommend”, to 7, “Would definitely recommend”.

We also used two open questions to gather parents’ perceptions and reflections on the program and its impact on them: “What was most memorable, useful and important for you?” and “Now that you’ve finished the group, what else would you like to share about the program, your experience?”

### 2.5. Data Analyses

SPSS27 (IBM Corp, New York, NY, USA, 2020) was used for quantitative analysis. Descriptive analysis of the demographic characteristics of participants and parents’ evaluation questions about the program was performed. To assess group differences (i.e., to compare parents who completed the program to parents who dropped out; parents who completed post-intervention assessment to non-completers), we applied the Chi square test for categorical variables and Mann–Whitney for age and PAFAS and MentS scores. To evaluate the changes in outcome variables (parenting, parental and family functioning, mentalization) from a baseline (pre-intervention) to post-intervention, we applied a paired samples *t*-test for PAFAS and MentS scores. To evaluate the effect sizes of these changes, Cohen’s d was calculated. The significance level was *p* < 0.05 for all analyses. 

For qualitative analysis, we analyzed answers to both open-ended questions about the program, in conjunction focusing on emerging themes. First, the initial analysis was performed to capture possible themes. The initial themes were discussed between authors (L.G.-P and R.B.), and material was analyzed again to decide on the final topics/categories. We categorized topics into two broader categories and subcategories with descriptions of the main aspect of each subcategory. When assigning answers to the “Mentalization” category, we used theoretical and clinical understanding and facilitation experience of the Lighthouse program, mentalization theory, assessment of mentalization, and mentalization-based therapy. Finally, we performed a final analysis assessing what subcategories emerge in every answer. The quotes from parents’ answers were used to illustrate categories and subcategories. The analysis was performed by the first and last authors. 

## 3. Results

**Figure 1:**
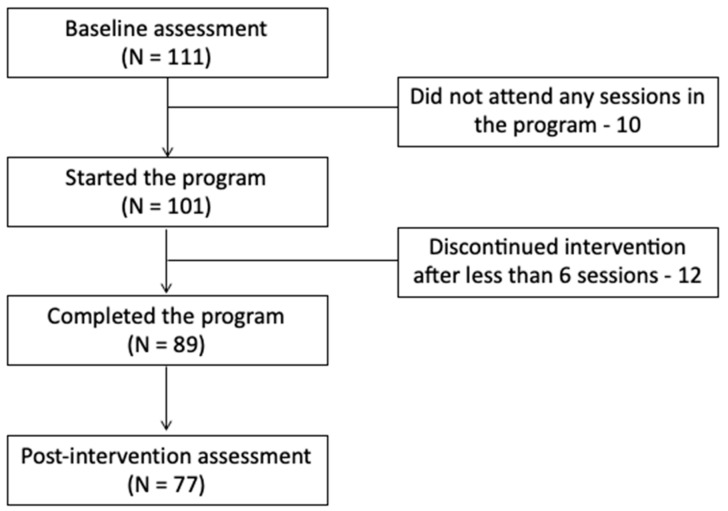
Study flow.

From 111 participants who initially registered for the program and filled in questionnaires at baseline, 101 participants started the program, 89 (88%) completed the program, and 77 filled in the post-assessment questionnaires (86.5% of participants who completed the program) (see Figure 1). 

First, we compared the demographic characteristics of parents who completed (*n* = 89) the program and who discontinued (*n* = 12). In most demographic characteristics, groups did not differ significantly, except in occupational status (a higher percent of employed parents completed the program, and a higher proportion of mothers on maternity leave discontinued the program). Comparisons of mentalization and parenting scores revealed no significant differences between completers and non-completers of the program. 

Secondly, we compared the demographics of parents who completed the program and filled in post-assessment questionnaires with the ones who completed the program but did not fill in the questionnaires after the program. Significant differences were found in terms of sex (a higher percentage of mothers completed and a higher percentage of fathers did not complete the post-assessment questionnaire) and education, where the difference was that the only participant who had lower-than-secondary education did not complete the post-assessment questionnaire. Interestingly, comparisons of mentalization and parenting scores showed that parents who had not completed the post-assessment questionnaire reported more positive encouragement pre-test (mean rank = 30.13) compared to the parents who completed the post-test assessment (mean rank = 46.77, U = 383.5, *p* = 0.034). 

Parenting and mentalization comparisons: Further, we compared parenting and family adjustment and mentalization scores at baseline with post-intervention scores (Table 1). The most significant change was in the decrease in coercive parenting practices with a large effect size (d = 0.86). Parents also reported significantly more positive encouragement with their children after the program (t = 3.73, *p* < 0.001, d = 0.43). Interestingly, the parental perception of parent–child relationship quality did not differ significantly after the program. In the family functioning domain, the scores improved significantly in all subscales. Parents’ emotional adjustment increased most significantly, with an effect size of 0.78. The general capacity to mentalize also improved after the intervention (t = −2.18, *p* = 0.033, d = −0.26). 

Parental evaluations of the program: The range in parents’ ratings on how beneficial the program ranged from 3 to 7 (7 being the highest score). Fifty parents (66.7%) rated the program as very useful, and 21% (*n* = 16) rated it a 6 (second-highest rating). Parents’ ratings of how likely they were to recommend the program to other people ranged from 3 to 7. The most common answer was “Definitely” (77.3%, *n* = 58), and 16.0% (*n* = 12) chose 6 (second-highest rating).

Parental experience and perceived benefit of the program (results from open-ended questions): Parents’ answers about what was important or what they wanted to share after the program contained a lot of positive feedback, with varied levels of detail in parental reflections about the experience or impact of the program on themselves or their children. The main emergent topics from what they shared were categorized into two broad categories, which we called “Mentalization” and “Parental experiences and functioning”, with three subcategories in each. These are presented in Table 2 with relevant quotes.

The “Mentalization” category represents parents’ answers where they directly mention any aspects of mentalization or there are indicators of mentalizing in their answers, or they indirectly write about mentalization, not referring to special terms or theory. The first subcategory (1) which emerged was the metaphors. Participants noted that metaphors helped them increase their understanding and analyze the behavior of their children and themselves better. Metaphors resonated with parents and were perceived as easily remembered and applicable in everyday life. The second subcategory (2) encompassed answers where parents indicated that psychoeducation about mentalization was important to them, or they noted their improved mentalization or described an improved mentalization process in themselves. The last subcategory was dedicated to parents’ answers on the importance of (3) understanding the connection between their past experiences and present relationships and reactions in parenthood. This link provided deeper insight into possible reasons for their reactions and, for some parents, showed that their past experiences can serve as a basis for the expanded understanding of their child.

We named the second broad category “Parental experiences and functioning”. Here, we assigned answers describing perceived impact on parental emotional state, parental self-view, or perceived changes in relationship with a child or in a family and important aspects of experience in the group. One of the subcategories within this domain touches upon self-reported parental changes in their improved emotional state and self-perception (subcategory 4). It includes mentions of improved well-being and changes in how parents view themselves in a parental role. Another subcategory (5) was defined as perceived changes in relationships or in attitude towards relationships. Here, parents shared what they noticed being different in their relationship with their children (e.g., less conflicts, more understanding, less excessive demands on children, etc.) or with other people (e.g., how they perceive relationship dynamics in the family or with significant others). The last category (6) was related more to experiences in the group. Many parents emphasized the importance of the shared experience; the fact that they are not alone with their troubles and concerns in parenthood, a sense of togetherness, and unity in the group came up a lot in their answers. The importance of the opportunity to share, to listen, and to be heard was also named by parents as being very important.

Moreover, quite a lot of parents shared that they felt the need for some kind of continuation of the program (e.g., parental self-support groups, recurring less-frequent meetings with facilitators, etc.). There were parents who experienced the 12-week program as being too short. They felt that they had just started feeling the therapeutic impact, were getting somewhere, were understanding what was offered in the program, and/or were establishing connections with the group, but the program stopped. Some of them shared that they anticipate the need to be reminded of LPP principles and the mentalizing position in future. One more common topic which emerged in the answers was parents’ view that the program should be offered to many more parents and be available more widely.

## 4. Discussion

The aim of this study was to evaluate whether parents’ mentalization, parenting, and level of parental and family adjustment changed after a 12-week group Lighthouse Parenting Program and to analyze the parental experiences of the program. Study results revealed that mentalization, parental adjustment, and family functioning improved, while coercive parenting practices decreased after the intervention. These results provide preliminary indications of the benefits of the 12-week Lighthouse Parenting Program group version for parents referred or self-referred for mental health services due to their own or their child’s difficulties.

Firstly, there was a significant decrease in coercive parenting practices after the program. Coercive parenting encompasses parental interactions with the child that can be harsh, negative, and hostile such as shouting, getting angry with the child, arguing with the child, using guilt and shame to teach a lesson, and using physical punishment for misbehavior [32]. The latter type of coercive behavior—corporal punishment—is closely linked with physical abuse [33]. Other aforementioned aspects of coercive parenting behaviors, even though they might not overlap with child maltreatment, are considered to be potentially physically or psychologically harmful [32] and detrimental to child development [34] in the long term. Through the lens of mentalization theory, on which LPP is based, the situations of hostile and harsh interactions can be considered a breakdown of mentalization [20]. Thus, the program focuses on instances during parent–child interactions where there is a high level of emotional arousal, and it is challenging for parents to mentalize. This is to help parents to recover the ability to reflect and react in a more controlled and flexible way; to regulate their emotions and behaviors in those situations. The decreased level of parental coercive behaviors after the program might be considered an important preliminary finding, as LPP aims to prevent harmful parenting.

In addition to that, the results show that, after the program, parents used more positive encouragement with their children when they behaved well. Even though the program does not specifically teach it, this might also indicate that the program increased parents’ understanding of the importance of seeing children when they behave well and to communicate to them that they are being noticed behaving well. This might suggest the impact of modeling: in the MBT group, facilitators aim to notice and label successful mentalizing [35] in parents, and this might add to the increase in similar behavior in parents. This can be further elaborated on via the grounds of the parents’ feedback, where they stressed their experiences in being inspired and feeling hopeful and stronger after the program. As such, this might add to the tendency for more use of positive encouragement.

Contrary to our expectations, the change in the quality of parent–child relationships was not significant. This finding can be anchored in the data provided in other similar studies. What can be seen from the studies on MBT parenting interventions is that most indicators of relationships are assessed through observational measures [19,22], and when the change is present, it is evident on the behavioral level in parents of small children. There are some indications of fewer negative interactions between mothers and their infants [36] and some improvements in parental behavioral sensitivity to the child’s cues [20]. In our study, parent–child relationships were measured by self-report, which provides an indication of perceived relationship quality. Moreover, most of the children were beyond early childhood years. Therefore, it is possible that the short self-report measure might not capture some relevant aspects of the relationships or deeper relational dynamics. In addition, from analysis of answers about the program, we see that parents reported perceived changes in their relationships with their child. This illustrates that some parents noted the improved connection after 12 sessions despite the insignificant difference on the group level. It is also possible that these changes take longer to be established and noticed by more parents. For example, one study found a more pronounced change in mother–child interactions after 12 months of follow-up [37], which may suggest that relationship changes might need time to unfold.

Moving on to the parents’ well-being, we measured overall emotional adjustment to the parenting role, which encompasses experienced levels of stress, depression, and anxiety [30]. Our results show that parental emotional adjustment level improved significantly after the program. This can also be illustrated by parents’ answers about noticing themselves as being calmer and reporting improved well-being. The review of mentalization-based parenting group programs found mixed evidence for reduced stress, anxiety, and depression after the groups [19]. In this context, our study aligns with other studies on MBT parenting programs where such change was observed (e.g., [38,39,40]). Our results are also in line with the results from the original version of the LPP, where reduced parental stress was reported [20]. Research on child maltreatment correlates shows that psychological distress can interfere with optimal parenting [41], and depression, anxiety [42], and parenting stress [7,43] are related with risk for child abuse. Thus, the decrease in dysfunctional emotional adjustment in our study might point to an important decrease in risk for suboptimal parenting or even childhood maltreatment.

Family functioning and a sense that parents work as a team increased after the program. Mostly only one parent from the family participated in the program, but it seems that, at least through the perspective of the participating parents, there was more support and positive interactions, fewer conflicts, and less criticism in families after the program. In those parents who were coparenting, the sense of parental teamwork improved. It might be possible that there was a change in how the participating parent interacted in the family during and after the program, which influenced family relationships.

Parenting practices, family functioning, and parental emotional adjustment are outcomes which are quite often included in studies of different parenting programs within the field of childhood maltreatment prevention. In general, our results showing the decrease in coercive parenting and increase in positive encouragement echo the effects of other parenting programs [11,13]. Regarding parental well-being and family functioning, the results are mixed, and modest effects of parenting programs have been provided [11,13]. Authors suggest that one possible explanation might be that the programs focus more on parenting skills, but not parental stress [13]. From this perspective, our study shows a promising increase in parental adjustment. This might be related to the LPP’s explicit focus on the impact of parental stress and emotional states when discussing possible mentalization impairments in high-arousal situations. The increased parental emotional adjustment might also be related to the experienced emotional support received from the group during the program (as indicated in parents’ feedback).

Finally, the LPP program is specifically tailored to increase parents’ capacity to mentalize, i.e., ability to understand behavior in terms of mental states [20]. Therefore, in addition to wider relevant outcomes, we aimed to assess whether parents’ mentalization improved. The results of the study show that the general level of mentalization increased after the program. The meta-analysis on MBT parenting interventions showed that there was an improvement in child-focused mentalization or mentalizing in a relationship context after interventions, with varied effect sizes [23]. Our study extends these results by revealing the increase in general capacity to mentalize, which encompasses self-oriented and other-oriented mentalization and the motivation to mentalize.

Furthermore, the importance of mentalization theory and change in mentalizing abilities emerged often in parents’ reflections on the program, with three separate subcategories. Firstly, it seems that metaphors were memorable and useful for parents. They served as an aid for mentalizing children and parents themselves. During the LPP, focus is also placed on increasing parents’ awareness that their current reactions and experiences in parenthood can be related to their past attachment or other significant interpersonal experiences. Parents’ answers illustrate that, at least for some parents, this was a very important insight which stood out for them. This whole category on mentalizing shows that the concept and experience of mentalizing made an impact on parents and reached them, not only cognitively but also emotionally.

The current study provides encouraging indications regarding the acceptability of the intervention by participants. Parents’ evaluation of the program was very positive, with most of them giving high ratings on the benefit and their probability of recommending the program to others. The retention rate can be also considered to be one of indicators of acceptability [44], and in our study, it was quite high—88 percent of parents who started attending the group finished the program. Additionally, while sharing their experience of the program, parents suggested that it should be available to other parents on a wider scale. Moreover, overall positive parental reflections on the program and in the group setting, emphasis on the sense of togetherness, and some parents’ answers strongly underlining their experienced importance of the program also point to the acceptability of the program to participating parents.

Our findings about parental experiences echo findings from a small sample of parents [20] where similar topics of increased self-confidence, decreased demandingness for oneself, improved understanding of children, and the sense that they are not alone emerged. This shows that parental experiences are similar in the shorter, group-only version of the program. Study results are relevant in the further implementation and development of the LPP. The original program is of 20 weeks long, with weekly group sessions and fortnightly one-to-one sessions with an individual therapist for high-risk families [20]. Our study provides preliminary evidence of the shortened version, the 12-week program [26]. It adds new empirical evidence on the scope of the program’s utility in different groups of parents, in our case, for parents facing parent–child relationship difficulties and mental health difficulties of their own or their children. Parental feedback and experience of the program, highlighting what was vital for them, can inform practitioners working with the program and other specialists working with parents on what they find important and impactful in the MBT parenting program.

### Limitations and Future Research Directions

Some limitations of the study and relevant future directions should be mentioned. The first limitation is the absence of a control group, which impacts the generalizability of the results. A study with a control group would be highly important in obtaining further results on the effectiveness or efficacy of the LPP. Moreover, self-report measures were used for parenting, family functioning, and mentalization. This means that we rely on information from one source—the parents themselves. When possible, behavioral measures on parent–child interactions, interview-based measures of mentalization, or the multi-method assessment of mentalization could allow for more reliable and wider understanding of possible outcomes. Studies with more assessments on mentalization and follow-up assessments could also analyze whether increased mentalization in parent–child relationships contributes to improved parenting practices providing important knowledge about possible mechanisms of change. One more limitation of our study is that we did not use one of the PAFAS scales—Parental Consistency—which showed low reliability, so this also should be investigated further in the Lithuanian sample.

## 5. Conclusions

The Lighthouse Parenting Program shows initial indicators of preliminary effectiveness and demonstrates that it might be a promising intervention in the prevention of childhood maltreatment with decreasing coercive parenting and increasing parental and family adjustment. One of the specific aims of the program is to increase parental mentalization, and the results are encouraging, as they provide some preliminary evidence that the program improves the general level of mentalization in parents.

## Figures and Tables

**Table 1:** Pre–post comparisons of parenting and mentalization.

		Pre	Post	t	df	*p*	d
		M (SD)	M (SD)				
PAFAS	Coercive Parenting	6.21 (2.43)	4.43 (2.01)	7.51	75	<0.001	0.861
	Positive Encouragement	3.20 (2.01)	2.59 (1.79)	3.73	74	<0.001	0.431
	Parent–Child Relationship	3.53 (2.92)	3.18 (2.54)	1.52	75	0.132	0.175
	Parental Adjustment	7.52 (2.97)	5.73 (2.70)	6.72	74	<0.001	0.776
	Family Relationships	4.84 (2.30)	4.36 (2.22)	2.15	74	0.035	0.248
	Parental Teamwork	3.97 (2.44)	3.41 (2.08)	3.00	65	0.004	0.369
MentS. Mentalization		107.96 (12.38)	110.45 (11.36)	−2.18	70	0.033	−0.258

**Table 2:** Categories and subcategories which emerged in parents’ answers about experience of the program.

Category	Subcategory	Explanation of Subcategory	Quotes
Mentalization	1. Metaphors	Any mention of metaphors used in the program (lighthouse, illuminating beam, safe harbor, piracy, raft, etc.)	“The metaphor of the lighthouse is often present in my everyday life; when it becomes difficult—I remember that the mission of the parents is to be a lighthouse”. “I really liked the metaphor of the lighthouse, and the whole story around it, about the raft, the piracy—it makes it easier to understand yourself and the child”. “I have gained knowledge, understanding and clarity about piracy and other forms of child behavior, where before I couldn’t understand what was going on and felt helpless”. “I really liked the use of metaphors. It helped me to observe and understand my feelings and behavior from the outside”.
	2. Understanding or indicators of mentalization	Mention of mentalization or mentalizing processes either in theoretical terms or mention of experienced aspects of mentalizing (e.g., understanding emotions, awareness, labeling, thinking about reasons underlying behavior, etc.)	“I have realized how important it is not to decide for the child and not to tell (not to convince) them how they feel (because our perceptions are influenced by our own experiences), but to be curious and interested without any preconceptions”. “Parents can relearn how to see the world through their child’s eyes”. “The meetings helped me to reflect, to calmly evaluate my relationship with my child, […]. Looking inward at myself, my childhood, discovering connections to the present and the experiences of other participants, where I also found a part of myself, helped my inner emotional state a lot. There were some ideas, which came to me anew during the meetings; it was like a discovery, which I am very happy about”. “To learn to feel, not only to get theoretical knowledge”. “It is important to understand yourself, to take care of yourself. To ask not only yourself how you feel, but also the child, to help the child understand how he feels and to be the person he does not fear, but wants and can come to in times of difficulty”. “I’ve learned that one can […] learn to lessen one’s own emotions in order to think clearly, and also to develop the skill of not falling into other people’s emotions, no matter how difficult they may be personally, to think about what they are about for me, what they mean to me, and why it is hard for me to bear them. Not just emotions, but also situations”. “That sometimes I am too certain that I know and understand my child. You have to keep being curious”.
	3. Reflection on the link between past and present	Insight that (how) past attachment experiences have impact on current parenting	“How much of our feelings, emotional states, experiences or traumas determine how we react to children’s behavior and emotions”. “Through several memories from my childhood, I realized how important it is for a child to be heard, how important it is not to touch a child if they don’t want to, to wait for the right time until they are ready to talk”. “That our feelings, reactions, and behavior are strongly influenced by our previous experiences and the way we were treated in childhood”. “To connect my childhood experiences with my current behavior with my children—this is probably the greatest gift of this program for me”.
Parental experiences and functioning	4. Perceived impact on parental self-perception or well-being	Mention of parents’ experience of a change in their own emotional state, in how they perceive themselves as parents	“I’ve become calmer, I trust my decisions in communication with the child”. “I have become more forgiving towards myself and my children”. “Thank you for an experience that, at least in the short term, has made a fundamental change in my life (both personally and as a mother). I now feel like I am standing pretty solidly on the ground. Although there is some anxiety about the future, I know the tools and means, what to do and where to turn if things get tough again”. “The meetings have inspired me, given me strength in my parenthood, and “recharged” me for a while ahead”. “Gave me confidence as a mother”.
	5. Perceived impact on parent–child relationships or parenting	Mention of perceived changes in relationships or changes in parental attitude towards relationships; changes in parental behavior in the family	“The arguments and raised tones with my daughter have decreased, and we talk more often and talk about how we feel”. “I no longer make very high demands on children”. “I feel I have a better relationship with my daughter; the idea of mentalizing helps to improve relationships in all areas of life”. “An important idea of mentalizing, I have started to put it into practice even more, and I have noticed a positive change in children’s behavior”. “It is important to get a reminder that it is normal to make mistakes when building a relationship, and if you make a mistake, to know that it is possible to repair the relationship”.
	6. Sense of togetherness, experience sharing	Mention of a chance to share and to listen to others share experiences, thoughts, feelings; feeling that you are not alone, sense of unity, togetherness	“The most important thing was a sense of togetherness in parenthood, when you can share experiences and learn from others”. “I really appreciated the openness of all the members of the group, the sharing of their different experiences, whether joyful, difficult or worrying; we all felt safe and were more or less heard”. “The sense of togetherness was very important and irreplaceable”. “The biggest impression in the group was made by other parents and their parenting experiences. It is wonderful to realize that although all of our parenting experiences are individual, we share and strive together for the well-being of our children”. “I couldn’t single out anything in particular as there was a lot of useful information, but I think the most useful thing was the psychotherapeutic group itself, when you realize that you are not the only one struggling with your child. The sense of belonging is very inspiring, and the different experiences and points of view are very optimistic that different solutions can still be tried”.

## Data Availability

The data presented in this study are available on request from the corresponding author.

## References

[1] Scully C., McLaughlin J., Fitzgerald A. (2020). The Relationship between Adverse Childhood Experiences, Family Functioning, and Mental Health Problems among Children and Adolescents: A Systematic Review. J. Fam. Ther..

[2] Baldwin J.R., Wang B., Karwatowska L., Schoeler T., Tsaligopoulou A., Munafò M.R., Pingault J.-B. (2023). Childhood Maltreatment and Mental Health Problems: A Systematic Review and Meta-Analysis of Quasi-Experimental Studies. Am. J. Psychiatry.

[3] Matjasko J.L., Herbst J.H., Estefan L.F. (2022). Preventing Adverse Childhood Experiences: The Role of Etiological, Evaluation, and Implementation Research. Am. J. Prev. Med..

[4] Mikton C., Butchart A. (2009). Child Maltreatment Prevention: A Systematic Review of Reviews. Bull. World Health Organ..

[5] van IJzendoorn M.H., Bakermans-Kranenburg M.J., Coughlan B., Reijman S. (2020). Annual Research Review: Umbrella Synthesis of Meta-Analyses on Child Maltreatment Antecedents and Interventions: Differential Susceptibility Perspective on Risk and Resilience. J. Child Psychol. Psychiatry.

[6] Dube S.R., Anda R.F., Felitti V.J., Croft J.B., Edwards V.J., Giles W.H. (2001). Growing up with Parental Alcohol Abuse: Exposure to Childhood Abuse, Neglect, and Household Dysfunction. Child Abus..

[7] Crouch E., Radcliff E., Brown M., Hung P. (2019). Exploring the Association between Parenting Stress and a Child’s Exposure to Adverse Childhood Experiences (ACEs). Child. Youth Serv. Rev..

[8] Neece C.L., Green S.A., Baker B.L. (2012). Parenting Stress and Child Behavior Problems: A Transactional Relationship Across Time. Am. J. Intellect. Dev. Disabil..

[9] Jones C.M., Merrick M.T., Houry D.E. (2020). Identifying and Preventing Adverse Childhood Experiences. JAMA.

[10] World Health Organization (2016). INSPIRE: Seven Strategies for Ending Violence against Children.

[11] Altafim E.R.P., Linhares M.B.M. (2016). Universal Violence and Child Maltreatment Prevention Programs for Parents: A Systematic Review. Psychosoc. Interv..

[12] Doyle F.L., Morawska A., Higgins D.J., Havighurst S.S., Mazzucchelli T.G., Toumbourou J.W., Middeldorp C.M., Chainey C., Cobham V.E., Harnett P. (2022). Policies Are Needed to Increase the Reach and Impact of Evidence-Based Parenting Supports: A Call for a Population-Based Approach to Supporting Parents, Children, and Families. Child Psychiatry Hum. Dev..

[13] Chen M., Chan K.L. (2016). Effects of Parenting Programs on Child Maltreatment Prevention: A Meta-Analysis. Trauma Violence Abus..

[14] Volkert J., Taubner S., Byrne G., Rossouw T., Midgley N. (2021). Introduction to Mentalization-Based Approaches for Parents, Children, Youths, and Families. Am. J. Psychother..

[15] Allen J.G., Fonagy P., Bateman A.W. (2008). Mentalizing in Clinical Practice.

[16] Fonagy P., Bateman A.W. (2007). Mentalizing and Borderline Personality Disorder. J. Ment. Health.

[17] Fonagy P., Target M. (1998). Mentalization and the Changing Aims of Child Psychoanalysis. Psychoanal. Dialogues.

[18] Fonagy P., Gergely G., Jurist L.E., Target M. (2004). Affect Regulation, Mentalization, and the Development of the Self.

[19] Lavender S.R., Waters C.S., Hobson C.W. (2023). The Efficacy of Group Delivered Mentalization-Based Parenting Interventions: A Systematic Review of the Literature. Clin. Child Psychol. Psychiatry.

[20] Byrne G., Sleed M., Midgley N., Fearon P., Mein C., Bateman A., Fonagy P. (2019). Lighthouse Parenting Programme: Description and Pilot Evaluation of Mentalization-Based Treatment to Address Child Maltreatment. Clin. Child Psychol. Psychiatry.

[21] Cicchetti D. (2013). Annual Research Review: Resilient Functioning in Maltreated Children—Past, Present, and Future Perspectives. J. Child Psychol. Psychiatry.

[22] Byrne G., Murphy S., Connon G. (2020). Mentalization-Based Treatments with Children and Families: A Systematic Review of the Literature. Clin. Child Psychol. Psychiatry.

[23] Lo C.K.M., Wong S.Y. (2022). The Effectiveness of Parenting Programs in Regard to Improving Parental Reflective Functioning: A Meta-Analysis. Attach. Hum. Dev..

[24] Midgley N., Sprecher E.A., Sleed M. (2021). Mentalization-Based Interventions for Children Aged 6–12 and Their Carers: A Narrative Systematic Review. J. Infant Child Adolesc. Psychother..

[25] Volkert J., Georg A., Hauschild S., Herpertz S.C., Neukel C., Byrne G., Taubner S. (2019). Bindungskompetenzen psychisch kranker Eltern stärken: Adaptation und Pilottestung des mentalisierungsbasierten Leuchtturm-Elternprogramms. Prax. Kinderpsychol. Kinderpsychiatr..

[26] Byrne G., Ruggiero M. (2018). Lighthouse Parenting. Mentalization Based Group Programme. Facilitator Guide.

[27] Sleed M., Fearon P., Midgley N., Martin P., Byrne G., Zywek L. (2021). The Supporting Parents Project: A Randomised Controlled Trial of the Lighthouse Parenting Programme.OSF.

[28] Neukel C., Bermpohl F., Kaess M., Taubner S., Boedeker K., Williams K., Dempfle A., Herpertz S.C., The UBICA-II consortium (2021). Understanding and Breaking the Intergenerational Cycle of Abuse in Families Enrolled in Routine Mental Health Services: Study Protocol for a Randomized Controlled Trial and Two Non-Interventional Trials Investigating Mechanisms of Change within the UBICA II Consortium. Trials.

[29] Gervinskaite-Paulaitiene L., Barkauskiene R. (2022). An Effectiveness Study of Mentalization Based Parenting Program “Light-house” in Lithuania. OSF.

[30] Sanders M.R., Morawska A., Haslam D.M., Filus A., Fletcher R. (2014). Parenting and Family Adjustment Scales (PAFAS): Validation of a Brief Parent-Report Measure for Use in Assessment of Parenting Skills and Family Relationships. Child Psychiatry Hum. Dev..

[31] Dimitrijević A., Hanak N., Altaras Dimitrijević A., Jolić Marjanović Z. (2018). The Mentalization Scale (MentS): A Self-Report Measure for the Assessment of Mentalizing Capacity. J. Pers. Assess..

[32] Day J.J., Hodges J., Mazzucchelli T.G., Sofronoff K., Sanders M.R., Einfeld S., Tonge B., Gray K.M., MHYPeDD Project Team (2021). Coercive Parenting: Modifiable and Nonmodifiable Risk Factors in Parents of Children with Developmental Disabilities. J. Intellect. Disabil. Res..

[33] Bor W., Sanders M.R. (2004). Correlates of Self-Reported Coercive Parenting of Preschool-Aged Children at High Risk for the Development of Conduct Problems. Aust. N. Z. J. Psychiatry.

[34] Prinz R.J., Sanders M.R., Shapiro C.J., Whitaker D.J., Lutzker J.R. (2009). Population-Based Prevention of Child Maltreatment: The U.S. Triple P System Population Trial. Prev. Sci..

[35] Fonagy P., Campbell C., Bateman A. (2017). Mentalizing, Attachment, and Epistemic Trust in Group Therapy. Int. J. Group Psychother..

[36] Sadler L.S., Slade A., Close N., Webb D.L., Simpson T., Fennie K., Mayes L.C. (2013). Minding the Baby: Enhancing Reflectiveness to Improve Early Health and Relationship Outcomes in an Interdisciplinary Home Visiting Program. Infant Ment. Health J..

[37] Suchman N.E., DeCoste C.L., McMahon T.J., Dalton R., Mayes L.C., Borelli J. (2017). Mothering from the Inside Out: Results of a Second Randomized Clinical Trial Testing a Mentalization-Based Intervention for Mothers in Addiction Treatment. Dev. Psychopathol..

[38] Midgley N., Cirasola A., Austerberry C., Ranzato E., West G., Martin P., Redfern S., Cotmore R., Park T. (2019). Supporting Foster Carers to Meet the Needs of Looked after Children: A Feasibility and Pilot Evaluation of the Reflective Fostering Programme. Dev. Child Welf..

[39] Salo S.J., Flykt M., Mäkelä J., Biringen Z., Kalland M., Pajulo M., Punamäki R.L. (2019). The Effectiveness of Nurture and Play: A Mentalisation-Based Parenting Group Intervention for Prenatally Depressed Mothers. Prim. Health Care Res. Dev..

[40] Sieverson C., Santelices M.P., Farkas C., Espinosa N., Muzard A., Gómez D. (2021). Effects of a Mentalization-Informed Group Intervention with Videofeedback for Mothers of Preschool Children. J. Infant Child Adolesc. Psychother..

[41] Giallo R., Cooklin A., Brown S., Christensen D., Kingston D., Liu C.H., Wade C., Nicholson J.M. (2015). Trajectories of Fathers’ Psychological Distress across the Early Parenting Period: Implications for Parenting. J. Fam. Psychol..

[42] Calvano C., Engelke L., Di Bella J., Kindermann J., Renneberg B., Winter S.M. (2022). Families in the COVID-19 Pandemic: Parental Stress, Parent Mental Health and the Occurrence of Adverse Childhood Experiences—Results of a Representative Survey in Germany. Eur. Child Adolesc. Psychiatry.

[43] Geprägs A., Bürgin D., Fegert J.M., Brähler E., Clemens V. (2023). Parental Stress and Physical Violence against Children during the Second Year of the COVID-19 Pandemic: Results of a Population-Based Survey in Germany. Child Adolesc. Psychiatry Ment. Health.

[44] Sekhon M., Cartwright M., Francis J.J. (2017). Acceptability of Healthcare Interventions: An Overview of Reviews and Development of a Theoretical Framework. BMC Health Serv. Res..

